# 

**Published:** 1904-11-19

**Authors:** 


### NOTICE TO CORRESPONDENTS.

All MSS., Letters, Books for Review, and other matters intended for the Editor should be addressed The Editor, "The Hospital," The Hospital Buildings, 28 & 29 Southampton Street, Strand, W.C.

The Editor cannot undertake to return rejected MS., even when accompanied by stamped directed envelope.

**Notice to Advertisers and Subscribers.**

All Advertisements, Orders for Copies of The Hospital, and Business Communications should be addressed to **THE MANAGER (Not to the Editor),** The Hospital, 28 & 29 Southampton Street, Strand, London, W.C.

The Cost of Subscription, post free, is as follows:—Per week, 3½d.; per quarter, 3s. 3d.; per half-year, 6s. 6d.; per annum, 13s. Foreign and Colonial:—Per annum, 19s. 6d., payable, in all cases, in advance.

**NOTICE.—Vol. XXXVI. of "The Hospital" (April to September 1904), in handsome cloth binding, is now on sale at the office of this Journal, price 10s. 6d. Cases for binding the Numbers contained in this Volume 2s. Index to Vol. XXXVI., 3½d., post free.**

**The Monthly part for November is now ready. Price 1s., by post, 1s. 3d.**

### Medical Appointments.

S. Jervois Aarons, M.D.Edin., M.R.C.P.Lond., Pathologist and Curator of the Museum Hospital for Women, Soho Square, W. Frank Barnes, M.B., B.S.Lond., F.R.C.S.Eng., Honorary Surgeon to the Royal Orthopædic and Spinal Hospital, Birmingham. Thomas R. Bradshaw, B.A., M.D.Dubl., F.R.C.P.Lond., Physician to the Liverpool Royal Infirmary, appointed Examiner in Medicine in the University of London. T. Coogan, M.B., Ch.B.Vict., Junior Resident Assistant Medical Officer, Chorlton Union Workhouse. William Henry Davis, M.B., B.Ch.R.U.I., House-Surgeon to the Royal Victoria Hospital, Belfast. D. L. Davies, M.D., Medical Officer of Health, Wisbech Rural District. Arthur L. Flemming, L.R.C.P., M.R.C.S., Anæsthetist, Royal Infirmary, Bristol. George T. Gifford, M.D.Durh., M.R.C.S. Eng., Honorary Assistant-Surgeon to the Blackburn and East Lancashire Infirmary. S. T. Irwin, B.A., M.B., Ch.B., Surgical Registrar to the Royal Victoria Hospital, Belfast. Hugh Lett, M.B., Ch.B.Vict., F.R.C.S., Assistant Surgeon to the Belgrave Hospital for Children. Frederic T. B. Logan, M.R.C.S., L.R.C.P., Medical Officer to Post Office (Bishopsworth Division), and Medical Officer under Elementary School Teachers (Superannuation) Act, 1898. M. M. Lyons, M.B., Health Officer for the Shire of Tungemah, Victoria. F. W. A. Margarey, M.D.Syd., Honorary Assistant-Surgeon to the Adelaide Hospital. William Moir, M.B., Health Officer for the Shire of Mirboo, Victoria. M. K. Moss, M.B., B.S.Melb., Junior Resident Medical Officer, Perth Public Hospital, West Australia. Arthur G. E. Naylor, L.R.C.P., Health Officer for the Shire of South Gippsland, Victoria. M. Ogilvy-Ramsay, M.D., F.R.C.S., Surgeon to the Cumberland Infirmary. Cecil E. Shaw, M.D., M.Ch.R.U.I., Lecturer in Ophthalmology in Queen's College, Belfast. Gerard C. Taylor, M.D.Cantab., D.P.H., County Medical Officer of Health for Berkshire. J. Thomson, M.B., Ch.B.Aberd., Medical Officer, Earlswood Asylum, Redhill.

**"The Hospital" Institutional Library.**

"Hospitals and Asylums of the World." 4 Vols., with a Portfolio of Plans, complete £12 12s.

"Burdett's Hospitals and Charities, 1904." 5s. net.

"Burdett's Official Nursing Directory." 8s. net.

"Cottage Hospitals, General, Fever, and Convalescent." 10s. 6d.

"Uniform System of Accounts for Hospitals and Public Institutions." New Edition. 4s. net.

"Hospital Expenditure: The Commissariat." 2s. 6d.

"A Legal Handbook for the Use of Hospital Authorities." 2s. 6d.

"The Cottage Hospital Case Book or Register of Patients." 10s. and 12s. 6d.

All these are published by the SCIENTIFIC PRESS, Ltd., and may be obtained through any bookseller, or direct from the publishers, 28 and 29 Southampton Street, Strand, London, W.C.

### Medical and Administrative Appointments.

THE HOSPITAL FOR SICK CHILDREN, GREAT ORMOND STREET, LONDON, W.C.

Notice is hereby given that the office of RESIDENT MEDICAL SUPERINTENDENT will become vacant on December 18th, 1904.

Candidates for this post are invited to send in their applications addressed to the Secretary, with copies of not more than three testimonials given specially for the purpose, on or before 12 o'clock on WEDNESDAY, December 7th, 1904.

Candidates must be registered medical practitioners, and must have held a responsible hospital appointment.

The appointment is made for one year, but may be held, subject to annual re-election, for a period of three years.

Salary 100 guineas per annum, with Board and Residence in the hospital, and £5 washing allowance.

All candidates must be present and appear before the Joint Committees at their meeting on Thursday, December 8th, 1904, at 5 P.M. precisely.

Forms of application and copy of rules may be obtained from the Secretary.

By order of the Managing Committee,
JAMES McKAY, Acting Secretary.

November 8th, 1904. (2112)

ADDENBROOKE'S HOSPITAL, CAMBRIDGE.

SECRETARY-SUPERINTENDENT WANTED.

He will be required to live close to the Hospital, and to devote his whole time and energies to its service. He will be responsible for all secretarial business, for the keeping of the accounts, for the collection and increase of subscriptions, and for the order and general administration of the Hospital in all matters except the medical and surgical treatment of patients.

He will be required to find security for £500.

He will be paid a commencing salary of £250 per annum. Clerical assistance will be given.

It is desirable that he should commence his duties on the 26th December, 1904.

All applications, which should be accompanied by not more than three testimonials, must reach the Secretary not later than the 3rd December, 1904.

Canvassing will be treated as a disqualification.

Particulars may be obtained from the Secretary.

JOHN BONNETT, Secretary.

23 St. Andrew's Street, Cambridge.
November 16th, 1904. (2111)

### BOOKS FOR

### Midwives & Maternity Nurses.

*Published by* THE SCIENTIFIC PRESS, LTD.

| | |
|---|---|
| **A COMPLETE HANDBOOK OF MIDWIFERY.** By J. K. WATSON, M.D. Containing over 350 pp., and Illustrated with 143 Diagrams. Cloth. | 6/- net, 6/4 post free. |
| **A PRACTICAL HANDBOOK OF MIDWIFERY.** By FRANCIS W. HAULTAIN, M.D. New Edition. Illustrated. Bound in leather. | 6/- net, 6/3 post free. |
| **NURSING NOTES ON MIDWIFERY AND GYNÆCOLOGY.** By SISTER ROSS, Rotunda Hospital. Suitable size for the apron pocket. Cloth. | 2/- net, 2/1 post free. |
| **THE MIDWIVES' POCKET-BOOK.** By HONNOR MORTEN. Cloth. | 1/- post free. |
| **HOW TO BECOME A CERTIFIED MIDWIFE.** By E. L. C. APPEL, M.B. Limp cloth. | 1/- net, 1/1 post free. |

***N.B.—All Forms and Books needed in accordance with the requirements of the new Midwives Act also Supplied.***

**The Scientific Press, Ltd.,** *Publishers and Booksellers of all Classes of Hospital Nursing Publications, Charts, Case Books, &c.*

**28 & 29 Southampton Street, Strand, London, W.C.**

**Complete Catalogue of Nursing Manuals post free on application.**

